# Health care-seeking patterns for female genital mutilation/cutting among young Somalis in Norway

**DOI:** 10.1186/s12889-018-5440-7

**Published:** 2018-04-18

**Authors:** Vivian N. Mbanya, Abdi A. Gele, Esperanza Diaz, Bernadette Kumar

**Affiliations:** 1 Department of Community Medicine and Global health, Institute of Health and Society, Faculty of Medicine, University of Oslo, P.O Box 1130, Blindern, 0318 Oslo, Norway; 2 Department of Nursing and Health Promotion, Faculty of Health Sciences, Oslo and Akershus University College of Applied Sciences, Oslo, Norway; 3 Norwegian Centre for Minority Health Research, Oslo, Norway; 4 Department of Global Public Health and Primary Care, University of Bergen, Bergen, Norway

**Keywords:** Female circumcision, Care-seeking, Immigrants, Somalis, Norway

## Abstract

**Background:**

Female genital mutilation/cutting (FGM/C) is a great concern, considering all the potential health implications. Use of health care services related to FGM/C by women who have been subjected to FGM/C in Norway remains to be understood. This study aims to explore the health care-seeking patterns for FGM/C-related health care problems, among young Somalis in Norway.

**Methods:**

A cross-sectional study involving 325 young Somalis in Oslo was conducted in 2014 using respondent-driven sampling (RDS) technique. The RDS was initiated by a small number of recruited seeds, who were given coded coupons to recruit their peers to participate in the study. Eligible recruiters who participated in the study and redeemed their coupons created the first wave of respondents. The first wave further recruited their peers, the second wave. The cycle continued to attain the needed samples. Using interviews and structured questionnaires, data on socio-demographic, FGM/C status and FGM/C-related use of health care were obtained. Logistic regressions were used to compute the odds ratio (OR) and the confidence interval (CI) for the associations between demographic variables, to circumcision status and health care-seeking for FGM/C. This study will focus on the 159 female participants of the total 325.

**Results:**

While 51.6% of the 159 women were subjected to FGM/C, only 20.3% of them used health care services for FGM/C-related problems. Women’s FGM/C status was associated with age ≥ 12 years at migration, experience of stigma regarding FGM/C practice (*p* <  0.05), support of FGM/C practice, and place of birth of women (*p* <  0.05).

**Conclusion:**

Only one-fifth of the women with FGM/C sought care for FGM/C-related health problems. Our study does not provide the answers to why only a few of them sought care for FGM/C. However, as a large proportion of women did not seek care, it is important to investigate the reasons for this. For, we propose to conduct further research targeting girls and women who have undergone FGM/C to assess challenges in accessing health care services for proper intervention.

## Background

Female genital mutilation/cutting (FGM/C) is a global health concern with numerous health and ethical implications. An estimated 200 million girls and women in 30 countries in Africa and some parts of Asia are affected by FGM/C. The practice is carried out mostly on young girls, between infancy and adolescence, with 3 million girls estimated to be at risk annually [1, 2]. FGM/C varies among countries, with Somalia, having the highest prevalence in the world [3]. Owing to globalized migratory processes, the tradition of FGM/C has spread to other countries, including countries in Europe and North America. Available national studies in the EU reveal prevalence rates for FGM/C among migrants of up to 49% [4].

According to WHO, FGM/C practice, “comprises all procedures involving partial or total removal of the external female genitalia or other injury to the female genital organs for non-medical reasons” [1]. There are four types of FGM/C: Type 1 involves the partial or total removal of the clitoris and the prepuce. Type 2 involves the partial or total removal of the clitoris and the labia minora. Type 3 (infibulation) is the most severe form, which involves the removal and the opposition of the inner and outer labia, with or without excision of the clitoris, leading to the creation of a covering seal and narrowing of the vagina opening. Type 4 incorporates all other harmful procedures to the genitalia [2]. The various forms of FGM/C can occur in different combinations and they are not practice to the same extent everywhere. The reason for the practice varies considerably between religion, ethnic groups, countries, and regions and the practice and the age at which girls are cut depends on the prevailing tradition of the area [5, 6]. While studies have demonstrated the immediate, obstetric, gynecological and psychological harmful health effects of FGM/C practices, long-term effects may vary [7–9]. The immediate complications involve hemorrhage, pain, shock, genital tissue swelling, infections, urination problems, and death. Other possible complications are sexual problems as dyspareunia, decreased sexual satisfaction, reduced sexual desire and arousal [10, 11]. A number of studies in Norway and abroad have shown the association between FGM\C and adverse obstetric outcomes, including episiotomy, prolonged labor, obstetric tears/lacerations and difficult labor/dystocia [12]. Following circumcision, women have been reported to suffer from post-traumatic stress disorder (PTSD), depression, loss of trust, lack of bodily well-being and permanent lifetime tissue damage [13].

Because FGM/C remains a pressing human right and public health issue, it is imperative for the societies and the international bodies to prohibit it by law [14, 15]. Performing any form of FGM/C in Norway is forbidden and punishable by law, with an incarceration term of up to 6 years and 15 years for severe cases [16]. However, according to the Norwegian Directorate of Immigration, many girls and women migrating from FGM/C practicing countries may have undergone FGM/C upon arrival in Norway [16]. There were 44,467 female immigrants from FGM/C practicing countries residing in Norway in 2013, of which 50% were estimated to have been subjected to Type 3 prior to migration [17]. A recent study in Norway indicates that 17,300 of women and girls who have undergone FGM/C can be in need of health care, [17, 18].

Health care-seeking behavior can be defined as a “sequence of remedial actions that individuals undertake to rectify perceived ill health” [19]. The Norwegian health system provides services for FGM/C care and FGM/C-related issues. Those who have undergone FGM/C, receive important information about the prohibition by legislation, the health consequences and health care provision related to FGM/C. They also receive the necessary health care and free treatment related to FGM/C [16]. Medical services are available to contact and talk about FGM/C and to get the right treatment. Women can contact their general practitioner, midwife/nurses at the local medical center or a school nurse. They can also contact the women’s or children’s clinic at their local hospital [16]. Women must be referred by their general practitioner (GP) to these specialized services. While health care provider’s experiences with women living with FGM/C during birth have been reported elsewhere [20], the health care-seeking pattern among women who have been subjected to FGM/C has not been explored. This study aims to explore the health care-seeking patterns for FGM/C-related health care problems, among young Somalis living in Norway.

## Methods

### Setting/study population

Participants were Somali women and men who took part in a cross-sectional study conducted in 2014 in Oslo using a respondent-driven sampling (RDS) technique. The RDS technique consists of an enhanced snowball sampling, in which information on who recruited whom and the individual social network size, provided the basis for the calculation of inclusion probability and minimally biased population indicators, as well as the variability of these indicators [21, 22]. The RDS was initiated by a small number of purposely recruited seeds, who were given uniquely coded coupons and incentives to recruit their peers to participate in the study. Eligible recruits who participated in the study and redeemed their coupons created the first wave of respondents. The first wave respondents were given incentives to further recruit their peers [21], who then formed the second wave, with the cycle continuing until the proposed sample size was achieved. RDS is largely used in behavioral studies [23], and it is widely acknowledged for its potential in studying immigrant communities whose sampling frame is unavailable [24–27].

### Inclusion criteria

Eligibility criteria for the study was being of Somali origin, permanently or legally residing in the Oslo municipality, between the ages 16 to 25 years, willing to participate and able to provide informed consent.

### Recruitment process

Four eligible seeds, comprising socially well-connected individuals, were selected based on a diversity of gender, location, years of stay in Norway and age. After providing informed consent, the seeds underwent an interview and were educated on how to further recruit other eligible young Somalis. They were given two uniquely coded coupons to help recruit their peers in their social network. The reason for using only two coupons was to elongate the recruitment waves so that the diversion of subsequent waves from the initial seeds was increased. Each seed proceeded to recruit two persons into their network, which became the first wave, with the first-wave participants further recruiting their peers, which then became the second wave. Each participant was compensated with 100 Norwegian kroner (NOK) for participating in the study and received an additional 100 NOK for each recruited peer who met the eligibility criteria and participated in the study. With 95% level of confidence and 5% margin of error, we obtained a desired sample size of 325. In this paper, the focus will be on the women participants.

### Variables

- FGM/C status was denoted as “yes/no” in reference to having been circumcised or not.
- Health care-seeking was denoted as “yes/no” in reference to having visited a GP or being referred for FGM/C health related issues.
- Support of FGM/C practice was denoted as “yes/no” in reference to participant’s opinion in encouraging the continuation or the discontinuation of the practice of FGM/C.
- Stigmatized by FGM/C practice was denoted as “yes/no” in reference to feeling stigmatized because of FGM/C practice.

### Data analysis

Descriptive statistics in the form of frequencies (percentages) and means with standard deviations were used to summarize the data at baseline. Chi-square tests and Fishers’ exact test (where appropriate) were used to establish an association between the FGM/C status and the socio-demographic variables. Generalized linear regression models (GLMs) with the logit function were used to fit binary responses relating to FGM/C and health care-seeking for FGM/C. Possible demographic factors that were associated with the response variables were identified and explained. The modeling process proceeded in two steps; first, simple GLMs were used to identify socio-demographics factors with *P* ≤ 0.05 for the FGM/C model. Secondly, variables that were significant (P ≤ 0.05) in the univariate analyses together with independent variables that have been previously shown to be associated with FGM/C were used to fit model 2. However, model 2 for the health care-seeking for FGM/C was based on independent variables with *P* ≤ 0.20. We used P ≤ 0.20 in order not to miss out important variables. Model 1 (full model) was adjusted for all socio-demographic factors. We used the Akaike information criterion (AIC) to select a better fit between model 1 and model 2. The AIC states that given a set of candidate models, the model with the smallest AIC estimate fits the data better. All analyses were performed using SPSS IBM Statistics 24 and the significance level was set at 0.05.

## Results

### Sample characteristics

The general characteristics of participants are summarized in Table 1. In this article, we have chosen to focus upon the results from the 159 women. Of the study participants, 51.1% were men and 48.9% were women, with a mean age of 19.7 (SD 1.90). Compared to the men, the women were slightly younger (19.3 vs. 20.0 years). The majority of the women (56.7%) had secondary education. 33.3% of the women were born in Norway while 66.7% migrated to Norway. The women’s mean age at migration was 11.07 (SD 4.7) years and 81.5% of them were single.

**Table 1:** Demographic characteristic of the study population

Variables	Male (*n* = 166)	Female (*n* = 159)	Total (*n* = 325)
Place of birth, (%)			
Born in Norway	44.6	33.3	39.2
Born out of Norway	55.4	66.7	60.8
Age, (mean ± SD)	20.0 ± 1.74	19.3 ± 1.98	19.7 ± 1.90
Age at migration to Norway, (mean ± SD)	13.43 ± 4.8	11.07 ± 4.7	12.20 ± 4.91
Marital status, (%)			
Single	62.5	81.5	71.7
Married	30.6	17.2	24.1
Divorced	6.9	1.3	4.2
Education, (%)			
University	16.9	13.4	15.2
Secondary	51.2	56.7	53.9
Primary	30.1	26.1	28.2
No formal education	1.8	3.8	2.8
Subjected to genital cutting, (%)	–	51.6	51.6
Support of FGM/C practices, (%)	7.3	10.8	9.0
Stigmatized of FGM/C practice, (%)			
No	67.7	79.1	73.2
Yes	32.3	20.9	26.8

### Association between FGM/C and socio- demographic characteristics

Among women in the study, 51.6% had been circumcised, while 48.4% were not circumcised. Using Chi-square statistic, significantly a higher proportion of the circumcised women were born out of Norway and were 12 years or older at migration as compared to those born in Norway and earlier age of migration respectively. Circumcised women more often supported the practice of FGM/C. No significant differences between women who were or were not circumcised were found regarding education level, marital statuses or stigma of the FGM/C practice, Table 2.

**Table 2:** Proportion of circumcised women across demographic variables

Socio- demographic characteristic of women	Women FGM/C status		Statistics	*P*-value
	Circumcised (*n* = 82)	Not circumcised (*n* = 77)		
Health care-seeking for FGM/C-related health problems	16 (20.3)	–	–	–
Place of birth:			X^2^ = 64.12	< 0.0001
Born in Norway	3 (3.8)	48 (64.9)		
Born out of Norway	76 (96.2)	26 (35.1)		
Age distribution (years):			X^2^ = 0.01	0.89
16 to 20	56 (69.1)	54 (70.1)		
21 to 25	25 (30.9)	23 (29.9)		
Age at migration to Norway			X^2^ = 37.82	< 0.0001
0 to 11 years	37 (45.1)	70 (90.9)		
≥ 12 years	45 (54.9)	7 (9.1)		
Education:			Fisher’s Exact	0.27
University	8 (10.0)	13 (16.9)		
Secondary	43 (53.8)	46 (59.7)		
Primary	25 (31.3)	16 (20.8)		
Informal education	4 (5.0)	2 (2.6)		
Marital status:			Fisher’s Exact	0.98
Single	64 (81.0)	59 (81.9)		
Married	14 (17.7)	12 (16.7)		
Divorce	1(1.3)	1 (1.4)		
Women in support of FGM/C:			X^2^ = 7.63	0.007
No	67 (82.7)	74(96.1)		
Yes	14 (17.3)	3 (3.9)		
Women stigmatized of FGM/C practice:			X^2^ = 1.14	0.28
No	59 (75.6)	62 (82.7)		
Yes	19 (24.4)	13 (17.3)		

Table 3 shows the odds ratios (OR) and their 95% CIs obtained from the binary logistic regression analyses. The unadjusted analyses showed that age at migration, support of FGM/C practice and place of birth were significantly associated with FGM/C. Based on the AIC, we present results from model 2. The analysis showed that participants who were at least 12 years when they migrated to Norway were almost 5-times likely to have undergone FGM/C compared with participants who were less than 12 years when they migrated to Norway (*P* = 0.01). We also observed that being born in Norway significantly reduced the odds of FGM/C by 98%.

**Table 3:** Logistic regression analyses for the associations between demographic variables and female genital mutilation/cutting

Female genital mutilation/cutting						
Demographic variables	Univariate analysis (unadjusted)		Model 1 (Adjusted)		Model 2 (Adjusted)	
			^a^AIC = 118.34		^a^AIC = 116.70	
	OR (95% CI)	*P* -value	OR (95% CI)	*P* -value	OR (95% CI)	*P* -value
Age						
16 to 20 years	1		1		1	
21 to 25 years	1.04 (0.53–2.06)	0.89	0.80 (0.23, 2.70)	0.71	1.20 (0.37, 3.84)	0.76
Age at migration to Norway						
0 to 11 years	1		1		1	
≥ 12 years	12.16 (4.99–29.62)	< 0.01	4.78 (1.53, 15.00)	0.01	4.84 (1.57, 14.91)	0.01
Marital status						
Single	1		1		1	
Married	1.07 (0.46–2.51)	0.86	0.86 (0.23, 3.21)	0.82	0.71 (0.20, 2.51)	0.60
Divorce	0.92 (0.05–15.07)	0.95	–	–	–	–
Support of FGM/C practice						
No	1		1		1	
Yes	5.15 (1.41–18.72)	0.01	2.06 (0.38, 11.10)	0.40	2.10 (0.39–11.43)	0.39
Education						
University	1		1		1	
Secondary	1.51 (0.57–4.02)	0.40	1.44 (0.32, 6.42)	0.64	1.73 (0.40, 7.43)	0.46
Primary	2.53 (0.86–7.48)	0.09	2.72 (0.48, 15.51)	0.26	3.06 (0.57, 16.38)	0.19
No formal education	3.25 (0.48–21.9)	0.22	10.34 (0.17, 618.48)	0.26	10.19 (0.46, 227.76)	0.14
Place of birth of women						
Born out of Norway	1		1		1	
Born in Norway	0.02 (0.01, 0.07)	< 0.01	0.01 (0.001, 0.10)	< 0.01	0.02 (0.005, 0.12)	< 0.01
Stigmatized of FGM/C practice						
No	1		1			
Yes	1.53 (0.69–3.38)	0.28	2.58 (0.59, 11.28)	0.21		

Model 1 is a full adjustment for all main demographic variables

Model 2 is based on variables that are statistically significant from the univariate analysis and variables that have been shown to be associated with FGM/C in the literature

^a^Model selection was based on the Akaike Information Criterion (AIC) which states that a model with a smaller AIC estimate fits the data better. Therefore, based on the AIC, model 2 was selected

### Health care-seeking for FGM/C

Out of the 51.6% subjected to FGM/C, 20.3% of the circumcised women accepted with a “yes” to have sought help for health problems related to FGM/C. Table 4 presents Chi-square statistics for the association of demographic variables and FGM/C health care-seeking. The odds ratios presented in Table 5 show the association between health care-seeking for FGM/C and socio-demographic factors. In the unadjusted analyses, age at migration to Norway and stigma of FGM/C practices were significantly associated with health care-seeking for FGM/C. Model 2 was also selected because it has a smaller AIC estimate. The odds for health care-seeking for FGM/C were 3.18 times higher for participants who had migrated to Norway when they were ≥ 12 years old compared with those who were < 12 years old (*P* = 0.05). The analysis also showed that stigma of FGM/C practices significantly increased the likelihood of health care-seeking for FGM/C by 5-times.

**Table 4:** Demographic variables and association to FGM/C health care-seeking

Demographic variables	Health care-seeking for FGM/C		Statistics	*p*-value
	Yes	No		
Place of birth:			X^2^ = 0.70	0.40
Born in Norway	0 (0.0)	3(4.8)		
Born out of Norway	14 (100)	59 (95.2)		
Age distribution (years):			X^2^ = 0.001	0.97
16 to 20	11 (68.8)	43 (68.3)		
21 to 25	5 (31.3)	20 (31.7)		
Age at migration to Norway			X^2^ = 0.02	0.87
0 to 11 years	7 (43.8)	29 (46.0)		
≥ 12 years	9 (56.3)	34 (54.0)		
Education:			X^2^ = 6.23	0.10
University	4 (25.0)	4 (6.6)		
Secondary	9 (56.3)	32 (52.5)		
Primary	3 (18.8)	21 (34.4)		
Informal education	0 (0.0)	4 (6.6)		
Marital status:			X^2^ = 3.04	0.21
Single	11 (68.8)	51 (85.0)		
Married	5 (31.3)	8 (13.3)		
Divorce	3 (0.0)	1 (1.7)		
Women in support of FGM/C:			X^2^ = 0.05	0.81
No	12 (80.0)	52 (82.5)		
Yes	3 (20.0)	11 (17.5)		
Women stigmatized of FGM/C practice:			X^2^ = 4.34	0.03
No	9 (56.3)	48 (81.4)		
Yes	7 (43.8)	11 (18.6)

**Table 5:** Logistic regression analyses association of demographic variables, in relation to FGM/C health care-seeking

Health care-seeking for FGM/C						
Demographic variables	Univariate analysis (unadjusted)		Model 1 (Adjusted)		Model 2 (Adjusted)	
			^a^AIC = 89.97		^a^AIC = 89.91	
	OR (95% CI)	*P* -value	OR (95% CI)	*P* -value	OR (95% CI)	*P* -value
Age at migration to Norway						
0 to 11 years	1		1		1	
≥ 12 years	3.25 (1.13, 9.34)	0.03	2.85 (0.87, 9.41)	0.09	3.18 (1.02, 9.98)	0.05
Marital status						
Single	1		1		1	
Married	2.54 (0.79, 8.13)	0.12	3.99 (0.98, 16.24)	0.053	3.43 (0.90, 13.17)	0.07
Divorce	–	–	–	–	–	–
Education						
University	1		1		1	
Secondary	0.49 (0.13, 1.80)	0.29	0.28 (0.06, 1.41)	0.12	0.35 (0.08, 1.61)	0.18
Primary	0.32 (0.06, 1.62)	0.17	0.18 (0.03, 1.24)	0.08	0.26 (0.04, 1.51)	0.13
No formal education	–	–	–	–	–	–
Stigmatized of FGM/C practice						
No	1		1		1	
Yes	3.75 (1.26, 11.14)	0.02	5.03 (1.33, 18.94)	0.02	5.10 (1.46, 17.79)	0.01
Age						
16 to 20 years	1		1			
21 to 25 years	1.10 (0.36, 3.36)	0.87	0.44 (0.10, 1.87)	0.27		
Support of FGM/C practice						
No	1		1			
Yes	2.14 (0.54, 8.53)	0.28	1.52 (0.29, 7.93)	0.62		

Model 1 is a full adjustment for all main demographic variables

Model 2 is based on variables that have *P* ≤ 0.20 in the univariate analyses

^a^Model selection was based on the Akaike Information Criterion (AIC) which states that a model with a smaller AIC estimate fits the data better. Therefore, based on the AIC, model 2 was selected

## Discussion

FGM/C remains a common practice in the countries where it is traditionally performed [28], but the tradition is seldom maintained after migration [29, 30]. Despite the worlds’ effort to discourage FGM/C, it remains a traditional norm deeply rooted within the culture and the tradition of the communities within the Horn of Africa [31–33]. In our study we found that of the total number of women with FGM/C in this study, fewer (20.3%) sought assistant for FGM/C-related health problems, although, some might have concealed they had sought treatment. The reasons for the fewer number of women using the health services are not understood, but, this could relate to a number of factors [20, 34]. Socio-cultural factors, associated with beliefs, expectations and values with regard to health, can be important in understanding the reasons for the fewer number of women using the health care services in the current study.

Based on the smallest AIC estimates in the current study, variations in FGM/C status across several characteristics were functions of the place of birth of women and age at migration with circumcised women predominantly among those born out of Norway and among those who migrated from the age of 12 years. FGM/C practice is unlikely to occur in Norway because migration has most likely brought a change in the FGM/C practice, due to the contextual effects of FGM/C such as its illegality, awareness, women’s educational status, women’s readiness for the discontinuity of the practice in Norway. Cases of FGM/C reported to the police between 2005 and 2016, were dropped because of lack of evidence and no criminal offenses were proven. To the best of our knowledge no case has been tried in the Norwegian courts so far [35]. Although our analysis reveals that women who supported the practice were more likely to be circumcised, it is noteworthy that the majority of the women who have undergone FGM/C are in support of the practice. Being circumcised may not necessarily mean being supportive of the practice [36]. Parents and family or the duress of social and peer pressure initiated the decision for circumcision in most cases [36, 37]. As most of the young women in the present study had been circumcised prior to migration, it is apparent that most of them were circumcised at an early age based on parental/family decision, as circumcision is a common practice between the ages of 6 and 8 years in Somalia [38]. Growing up in a new environment such as Norway, one would expect that with empowerment and awareness of FGM/C practice over time brings about change that leads the disapproval and discontinuation of the practice.

### Health care-seeking

According to AIC estimates, women’s age at migration and the stigma of FGM/C practice were associated with health care-seeking for FGM/C among girls and women who have undergone FGM/C. In our study, we cannot determine which health care service is mostly used for FGM/C, or the reasons why only 20.3% of women who have undergone FGM/C have sought care. However, given the lower proportion of women using health care services, might indicate a lack of need or it could be attributed to women’s perceptions and challenges of care [20, 39].

According to the health belief model, an individual’s state of readiness to take action for a health condition is determine by four dimensions. Firstly, the perceived susceptibility to the condition and the probable severity of the condition (defined in terms of physical harm or interfere with social functioning). Secondly, the perception of benefits associated with actions to reduce the level of threat or vulnerability. Thirdly, the assessment of potential barriers, including physical, psychological and financial barriers, and finally, the general health motivations triggering appropriate health behavior, including internal cues as symptoms and external cues like interpersonal interaction and communication [40–42]. Addressing the above concepts of the health belief models both for the women and the health care system could enhance the number of people who use the health care services and assessing the coverage of health services is important to determine the quality of health care services [43].

According to the Norwegian law, it is the duty of parents to ensure that their daughters who have undergone FGM/C receive the necessary care [16]. In as much as FGM/C has a complex socio-cultural perspective, a decision to seek care for FGM/C-related issues is contextual. In an African context, as FGM/C is associated with culture the decision to seek care lies with the woman herself, her husband and/or relatives. In some settings, health seeking for FGM/C may depend on the availability and the skills of care providers, illness characteristic (recognition and severity), the status of the woman, previous experiences and perceived quality of care [39, 42, 44].

Previous studies have reported FGM/C not to be optimal among migrants in receiving countries. This might be attributed to unskilled care providers in host countries, type of services offered, women’s experiences, patients-caregivers interaction and sometimes the costs of care [39, 44, 45]. Health care-seeking may also be affected by a state of vulnerability and traumatization. Care seeking for FGM/C may either enhance or lessen if the women feel embarrassed about their condition, feel inadequate, shy and tense. Revisiting the health services is possible if women were satisfied with the care they received, or their past experiences [46].

According to prior studies, Somalis, Eritrean and Sudanese women with FGM/C in Norway and Sweden, have complained of insufficient attention, support, lack of respect, lack of interpreters, neglect from health care providers and they were uncomfortable with the unpleasant and hurtful comments from caregivers during delivery [20, 34, 47]. Somali women in England, USA and Australia had expressed the feeling of humiliation and avoided questions from health care providers that triggered flashbacks [48, 49]. Other women with FGM/C were very concerned with the breach of their privacy and confidentiality [50]. It is often presumed that it is mandatory for health professionals to inform circumcised women of the care they should expect. However, in Norway, circumcised women complained of lack of information regarding what to expect during delivery [34].

While our data cannot provide the explanations for the findings it is highly likely that the women in our study lack the information and knowledge about access, availability and functioning and navigation of FGM/C related health care services in Norway. Therefore, challenges of care may not only affect the women who are new to Norway, but also those who have been living here for a long period. Furthermore, communication can be a potential barrier to care seeking. Because 96.2% of the circumcised women were born out Norway, language can hinder their access to seeking care, as language and poor communication have been reported by care providers and women to be a barrier to effective care [39, 51]. Lack of a well-functioning referral system [52] and a good social support network may also influence access to care. A good support network has been shown to empower FGM/C affected women in accessing antenatal and intrapartum services in England [48]. While some barriers are potentially easier to overcome, there remain some serious concerns regarding the perceptions and experiences of FGM/C affected women that calls for further research.

A step towards health care-seeking might be a step in the direction of the discontinuation of FGM/C, as 85.7% of FGM/C affected women from Somalia, have previously indicated their interest in the discontinuity of FGM/C practice [53]. We observed FGM/C prevalence of 51.6%, in our study indicating a significant decline among the younger age groups, compared to a higher prevalence of 79.3% among older age group in a prior study [17]. This is consistent with other reports of the discontinuation of FGM/C in Somali communities, both at home and abroad [50, 54, 55]. Readiness to seek help might indicate that these women trust the patient-centered care and/or the Norwegian health care system and that the health personnel are responsive to their needs and values. It could also indicate that the health care centers/ support centers might provide solace as well as access to information and educational resources.

Although fewer numbers of women used the health care services, it is worth highlighting that there was a significant association between women who felt stigmatized by the practice and health care-seeking. Although stigmatization has often been pointed out as a deterrent to seeking medical care among FGM/C affected women [9, 47, 56, 57], it is noteworthy stigma was not a barrier to health care-seeking in the present study. As most (56.7%) of these women have at least secondary education, they may have acquired knowledge of the negative effects of the practice through awareness campaigns in Norway. Good knowledge along with willingness to seek care despite the stigma of the practice could indicate changing attitudes and perceptions towards FGM/C. It may also indicate a readiness for compliance to address their health needs [58].

The age at migration to Norway influenced women’s use of FGM/C health care in our study. Those who arrived at a young age were less likely to seek care, one would expect that they may not need care or may not know if the services are provided. Even if they are aware of the services, they may be faced with difficulty in navigating the health care system. The fear of criminalization has been previously documented as a limitation to accessing much needed quality health services in host countries [9, 57]. However, we are unable to study this association in our study population as most of the affected women were circumcised prior to migration and we did not ask any direct questions related to the fear of criminalization as being a barrier to health seeking.

### Strength and limitation of the study

The strength of the study lies in using the RDS methodology, pretesting of data collection tools, recruitment of field workers trusted by the Somali community and adequate training of the field workers. Because the principal investigator and the data collectors spoke the same language, this rules out bias due to translation. While it is impossible to rule out selection bias using our sampling methods, nonetheless, the findings provide valuable information about those seeking care for their health problems. However, our study is limited in providing explanations as to why this is the case. We have not ascertained information such as the degree of FGM/C or the symptoms and that limits our interpretations.

## Conclusion

We have found that the proportion of women seeking care for FGM/C-related health problems is very low compared to the number of women with FGM/C but our study is unable to provide the explanations as to why this is the case. Therefore, it would be desirable to conduct further research targeting girls and women who have been subjected to FGM/C to investigate access problems for FGM/C health care-seeking. The insights gained are likely to be valuable and can serve as baseline information for further research and appropriate interventions for health seeking.

## Abbreviations

AIC: Akaike Information Criterion
CI: Confidence Interval
FGM/C: Female genital mutilation/cutting
GLMs: Generalized linear regression models
GP: General Practitioner
NOK: Norwegian Kroner
OR: Odds Ratio
PTSD: Post-traumatic stress disorder
RDS: Respondent-Driven Sampling
REK: Regional Committee for Medical and Health Research Ethics
SD: Standard deviation
SSD: Social Science Data registry
WHO: World Health Organization
